# Prevalence of large vessel occlusions in an unselected hospital-based stroke cohort in Sweden

**DOI:** 10.3389/fneur.2025.1549537

**Published:** 2025-03-13

**Authors:** Mihae Roland, Ioanna Markaki, Fabian Arnberg, Stefanos Klironomos, Christina Sjöstrand

**Affiliations:** ^1^Department of Clinical Sciences, Danderyd Hospital, Karolinska Institutet, Stockholm, Sweden; ^2^Department of Clinical Neuroscience, Karolinska Institutet, Stockholm, Sweden

**Keywords:** stroke, ischemic stroke, middle cerebral artery, thrombotic stroke, large vessel occlusion

## Abstract

**Introduction:**

Determining the prevalence of large vessel occlusions (LVOs) is important for planning and accessing mechanical thrombectomy treatment. Previous estimates vary greatly in studies, which might be related to different inclusion criteria and/or selection bias. In this cohort study, we aimed to determine the presence of LVO in an unselected, i.e., untriaged, hospital-based stroke cohort in Sweden.

**Methods:**

Stroke patients treated at Karolinska Huddinge University Hospital were consecutively collected during the years 2008 through 2015, identified by an ICD-10 diagnosis of ischemic stroke (I63). Patients with LVO were selected through radiology reports indicating LVO.

**Results:**

A total of 3,152 consecutive patient events had received a diagnosis of ischemic stroke during the study period. A total of 356 occlusion events were found in the internal carotid artery, the first two segments of the middle cerebral artery (M1, M2), and anterior cerebral artery (A1, A2), the vertebral artery, basilar artery and the first two segments of the posterior cerebral artery (P1, P2). This resulted in an LVO prevalence of 11.3% in this cohort. Seventy-six percent of occlusions were located in the anterior circulation, and 24% in the posterior circulation. The most frequent occluded vessel was M1 (*n* = 166, 39%).

**Conclusion:**

In this study of consecutively collected stroke patients the prevalence of LVO was lower compared to other studies, possibly related to our unselected patient cohort, limited use of CT angiography, and/or the LVO definition used. These results are of importance for decision-making regarding the capacity of comprehensive stroke centers.

## Introduction

Large vessel occlusions (LVOs) are occlusions in the proximal arteries of the cerebral circulation, associated with more severe outcomes compared to stroke patients without LVO (1, 2). Mechanical thrombectomy, the endovascular removal of the thrombus, is the gold standard treatment of large vessel occlusions and is typically available in most comprehensive stroke centers (3). Due to the need for specialized equipment and personnel, this procedure is available only at selected hospitals. Previous LVO estimations vary widely, ranging between 13 and 52%, depending on the definition and method, with few estimates from unselected patients (4, 5). A recent review suggests an LVO prevalence of 21% in patients with suspected acute ischemic stroke and an LVO prevalence of 30% in patients with confirmed acute ischemic stroke (4).

The aim of this study was to determine the prevalence of LVOs in an unselected hospital-based stroke cohort. A secondary aim was to determine whether LVO is more prevalent in patients below 65 years of age.

## Methods

We designed a hospital-based register on cerebrovascular diseases based on a standardized protocol including demographic characteristics, comorbidities, clinical investigation results, neuroimaging data, complications, and outcome (6). At the time of the study, the region consisted of six Primary Stroke Centers and one Comprehensive Stroke Center (Karolinska University Hospital in Solna). We studied patients admitted to the Primary Stroke Center of Karolinska University Hospital in Huddinge between 2008 and 2015, thus before the implementation of the prehospital Stockholm Stroke Triage System (7). At that time, the hospital had approximately 800 beds, serving an ethnically diverse population of around 250,000 persons. All medical records, including previous and current medication and laboratory results, are digitalized. This stroke cohort consists of a consecutive series of patients admitted to the nearest hospital at symptom onset and with a final main diagnosis of ischemic stroke (ICD-10: I63) (8).

In total, 3,152 ischemic stroke events were identified in the data registry between 2008 and 2015. All patients within this cohort had undergone computed tomography (CT) of the brain. In addition, CT angiography of the neck and proximal intracranial arteries was performed in 1547 (49.1%) patients within the acute phase of the stroke. The prevalence of LVOs within this stroke cohort was derived from the database by selecting all patient-events with a verified ischemic stroke diagnosis as the denominator and all events where a radiology report indicated the presence of an LVO as the numerator.

Neuroradiology reports that matched the following prefixes were retrieved by exhaustive text search: “dense-” (followed by vessel or media) or “thromb-” as well as the phrases: “hyperdense” or “occlusion.” Additional search criteria excluded phrases preceded by negation. Subsequently, 895 examinations from radiology results were then evaluated manually by reading the reports. If the same patient was affected by several events of LVOs demanding acute hospitalization on different occasions during the included years, these events were counted as separate LVO events. The same stroke-event could consist of occlusions in more than one vessel territory (Table 1). To avoid measurement bias, and in cases where the presence of symptomatic occlusion was unclear (e.g., high-grade stenosis) the scans were assessed manually by a neuroradiologist and a stroke neurologist.

**Table 1:** Occlusion location.

*N*	428	
Vessel	*n* (%)	Female sex (*n*, %)
Anterior	327 (76.4)	156 (47.7)
A1	8 (1.9)	5 (62.5)
A2	2 (0.5)	1(50.0)
ICA	83 (19.4)	30 (36.1)
M1	166 (38.8)	90 (54.2)
M2	68 (15.9)	30 (44.1)
Posterior	191 (23.6)	37 (19.4)
P1	18 (4.2)	6 (33.3)
P2	11 (2.6)	2 (18.2)
Vertebral	55 (12.9)	23 (41.8)
Basilar	17 (4.0)	6 (35.3)

A1, first segment of the anterior cerebral artery; A2, second segment of the anterior cerebral artery; ICA, internal carotid artery; M1, first segment of middle cerebral artery; M2, second segment of the middle cerebral artery; P1, first segment of the posterior cerebral artery; P2, second segment of the posterior cerebral artery.

Since there is no standardized definition of LVO, results are presented according to the most frequently used definition, which includes artery segments commonly targeted during thrombectomy (4). In the anterior circulation this includes the internal carotid artery (ICA), the two first segments of the middle cerebral artery (M1, M2), and the two first segments of the anterior cerebral artery (A1, A2). In the posterior circulation this includes the vertebral artery (VA), the basilar artery (BA), and the two first segments of the posterior cerebral artery (P1, P2). There were no missing data regarding occlusion location.

Demographics and comorbidities on all included patients were retrieved from the patient journal and stratified and presented in order of the most prevalent ICD-10 diagnosis.

The prevalence of LVOs was also investigated on a year-by-year basis. A trend was calculated by regression analysis.

### Statistics

The stroke cohort and the LVO cohort, respectively, were described with descriptive statistics regarding age, sex and comorbidities. Occlusion location was presented with number and frequency (*n*, %). Linear regression was used for the trend of prevalence during the study years. Chi-square test was used for determining association with LVO within age groups, with a pre-specified significance limit of *p* < 0.05. Analysis was performed in *R*-studio v. 2021.09.0.

## Results

In total, 3,152 patient-events received an ischemic stroke diagnosis, and 356 (11.3%) LVO events were identified.

Linear regression analysis of the annual prevalence, showed a significantly increasing trend of LVO events identified, with approximately 4.4 additional LVO events per year (*p* < 0.05^***^).

Seventy-six percent of the occlusions were located in the anterior circulation, 24% in the posterior circulation. The most frequent occluded vessel was M1 (*n* = 166; 39%); see Table 1.

**Figure 1:**
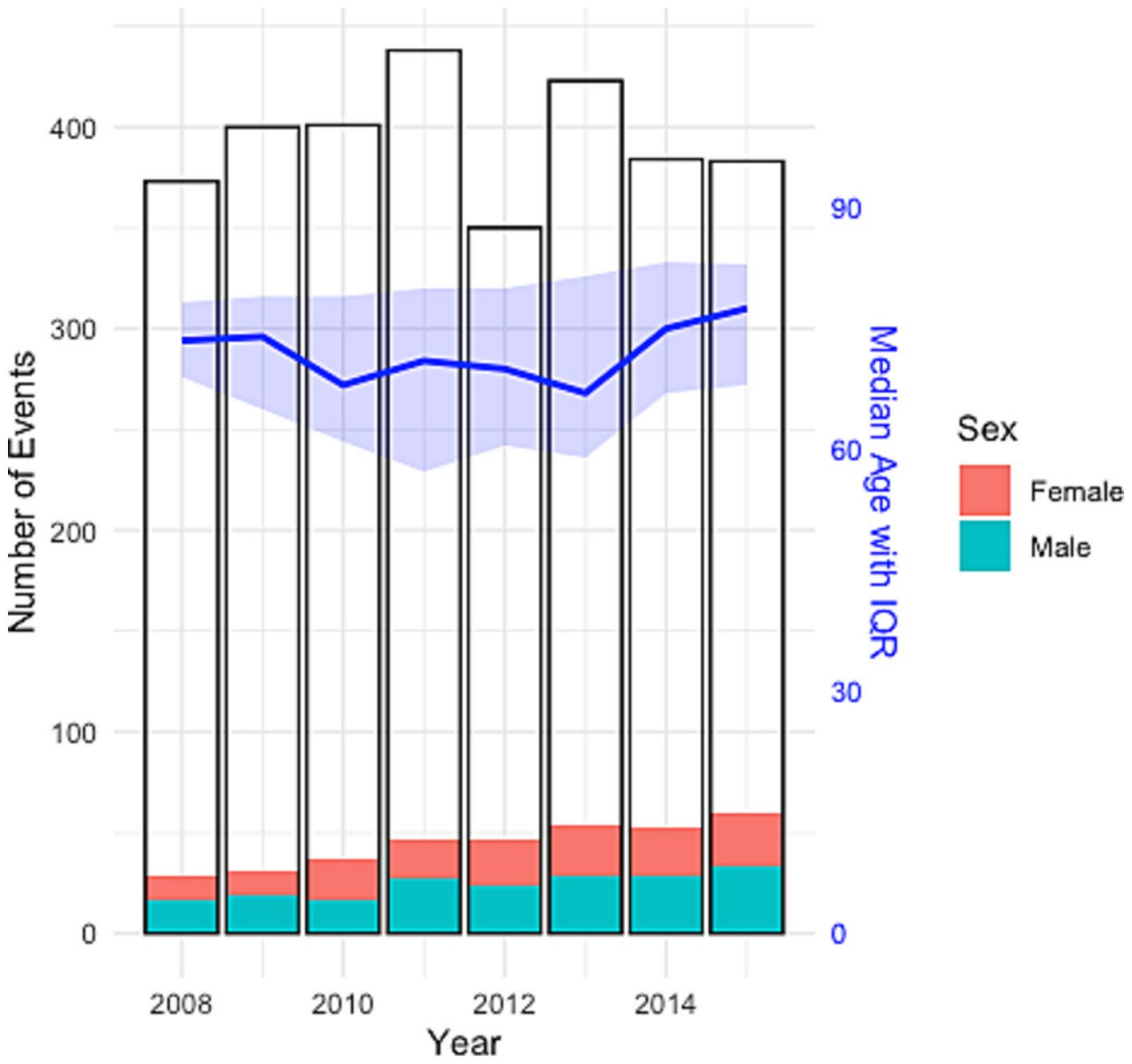
Sex distribution and median age of LVO events, with total number of ischemic stroke events per year.

Within the LVO cohort, 156 (45%) patients were females and median age was [age (IQR)] 73 [65–83]. Female stroke patients presented with LVOs later in life than male patients [78 (67–84) years vs. 69 (62–79); *p* < 0.05^***^]. The most common comorbidities for LVO patients were hypertension (*n* = 196, 57%), followed by atrial fibrillation (*n* = 122, 35%), diabetes mellitus (*n* = 73, 21%) and hypercholesterolemia (*n* = 65, 19%), see Table 2. Men had a higher prevalence of comorbidities in both the LVO cohort and the overall stroke population, except for atrial fibrillation, which was more common among women in the stroke group. There was no difference between the prevalence of LVO within ages below 65 (*N* = 94, 27.3%) and ages 65 and above (*N* = 685, 22.9%), *p* = 0.05. Figure 1 and Table 3 show the sex distribution and median age of LVO events.

**Table 2:** Comorbidities with sex distribution in LVO cohort and total stroke cohort.

Comorbidities	LVO patients		All stroke patients	
	*n* (%)	Female sex, *n* (%)	*n* (%)	Female sex, *n* (%)
Hypertension	196 (57)	88 (45)	1736 (58)	801 (46)
Atrial fibrillation	122 (35)	60 (49)	890 (30)	470 (53)
Diabetes	73 (21)	27 (37)	641 (21)	275 (43)
Hypercholesterolemia	65 (19)	23 (35)	498 (17)	211 (42)

LVO, large vessel occlusion.

**Table 3:** Prevalence of large vessel occlusions per year.

Year	*N*_events_/*N* (%)	Female sex, *n* (%)	Age (median [IQR])	Age < 65 years, *n* (%)
2008	29/373 (7.8)	12 (41.4)	73.50 [69.00–78.25]	6 (21.4)
2009	31/400 (7.8)	12 (38.7)	74.00 [65.00–79.00]	7 (22.6)
2010	37/401 (9.2)	20 (54.1)	68.00 [61.00–79.00]	12 (33.3)
2011	47/438 (10.7)	20 (42.6)	71.00 [57.25–80.00]	15 (32.6)
2012	46/350 (13.1)	22 (47.8)	70.00 [60.50–80.00]	13 (30.2)
2013	54/423 (12.8)	25 (46.3)	67.00 [59.00–81.50]	23 (43.4)
2014	52/384 (13.5)	24 (46.2)	75.00 [67.00–83.25]	10 (20.4)
2015	60/383 (15.7)	27 (45.0)	77.50 [68.00–83.00]	8 (14.0)

## Discussion

In this study, we aimed to determine the LVO prevalence in an unselected hospital-based ischemic stroke cohort, as well as whether patients of working age (<65 years) had more LVOs compared to older ages. We found an LVO prevalence of 11.3% in the ischemic stroke cohort, mainly located in the anterior circulation (76%), increasing with approximately 4.4 additional LVO events per year. Patients aged <65 years did not have more LVOs compared to older ages.

The LVO prevalence in this study aligns with a study reporting an LVO incidence of 11.1%, or 24 per 100,000, and a review article presenting a conservative estimation of LVO prevalence to 10–20% of all ischemic strokes (9, 10). However, our LVO prevalence is lower than those found in other studies, using the same broad LVO definition (23–52%) (11–16). The reason for this discrepancy might be related to differences in patient selection and methods, particularly the limited use of CT angiography in our study. Additionally, most studies introduced patient selection using pre-specified time constraints (12–15) or included patients referred from other hospitals (11). Furthermore, comorbidities may differ between stroke populations and affect the prevalence of LVO. In Sweden, the increasing use of direct oral anticoagulants as a prophylactic treatment for atrial fibrillation resulted in a decline in atrial fibrillation-related strokes between 2010 and 2020 (17).

Although LVO prevalence was low, we observed an increasing trend of LVO events per year, despite overall stable stroke rates, which could suggest increased detection. In relation to our lower LVO prevalence and limited use of CT angiography, more efficient and affordable examinations guide the clinical practice towards enhanced LVO detection (16). Moreover, estimations of LVO rates based on large stroke registries predicts rapidly increasing LVO incidences in the coming years, mostly driven by an aging population (18, 19).

The sex distribution of LVO locations in this study differs most in terms of anterior vs. posterior occlusions. In our sample, men had four times as many occlusions in the posterior circulation, in particular P1, P2 and basilar occlusions. While some studies suggest a higher proportion of posterior occlusions in men (20–23), others do not support this association (24, 25). Posterior occlusion strokes were seen to be associated with large artery atherosclerosis, compared to anterior circulation stroke where cardioembolism are more frequent (26). Amongst comorbidities, men were more prevalent in diabetes, hypercholesterolemia and hypertension in the LVO cohort. However, atrial fibrillation rates between sexes were similar. Interestingly, in the anterior circulation, women had slightly more M1 occlusions than men, as previously described (27, 28).

Stroke patients of working age risk permanent or long-term disability and potential economic loss from work, which in turn affects many aspects of their individual lives and society. We did not detect any statistically significant difference in prevalence rates of patients with LVO below vs. above the age of 65. Although LVO in young is expected to present less frequently as it is linked to vascular risk factors seen to increase with age, estimates of ischemic stroke rates in younger ages are on the rise (29) with an LVO prevalence of 18% in young ischemic stroke patients (aged 18–50 years) (30). Furthermore, although sex disparities are seen to diminish within the Swedish stroke population, women still present with stroke later in life compared to men, which was also confirmed in our cohort (31).

Determining LVO is important as it can inform decisions on access to reperfusion treatment. In Sweden, until recently, there was no nationwide registry reporting the number of LVOs in stroke patients. For reference, our LVO prevalence aligns with recent data on thrombectomy-treated patients in this region, that is 10% of all ischemic stroke (8% nationwide) (32). Notably, thrombectomy rates do not fully correspond to LVO prevalence. On one hand, procedures are sometimes performed on more distal segments of the cerebral vessels than those included in this study. On the other hand, in a clinical setting, some patients’ LVOs are left undetected, some have limited access to mechanical thrombectomy or are excluded from treatment due to other reasons, and some are successfully treated solely with intravenous thrombolysis.

### Limitations

The identification of LVOs in this study was determined by radiological assessment. Consequently, there might have been radiological reports where the key words were not used in which these cases might have been involuntarily excluded. In addition, half of the patients did not undergo CT angiography in the acute stage, based on clinical presentation and/or age or other contraindications. There is a possibility that there were unidentified LVOs in these patients as well.

## Conclusion

In this study of consecutively collected stroke patients the prevalence of LVO, 11.3%, was lower compared to other studies, although in line with recent thrombectomy rates in the region. Determining the prevalence of LVO is useful for guiding decisions regarding thrombectomy access and other aspects of stroke care planning.

## Data Availability

The data analyzed in this study is subject to the following licenses/restrictions: according to the ethical approval statement, the data that support the findings of this study are not publicly available as they contain information that could compromise the privacy of research participants. The data can be made available for qualified researchers from corresponding author MR upon reasonable request. Requests to access these datasets should be directed to mihae.roland@ki.se.

## References

[1] Nedeltchev K Schwegler B Haefeli T Brekenfeld C Gralla J Fischer U. Outcome of stroke with mild or rapidly improving symptoms. Stroke. (1970) 38:2531–5. doi: 10.1161/STROKEAHA.107.482554 17673713

[2] Zhu WMDP Churilov LP Campbell BCVPF Lin MB Liu XMDP Davis SMMDF. Does large vessel occlusion affect clinical outcome in stroke with mild neurologic deficits after intravenous thrombolysis? J Stroke Cerebrovasc Dis. (2014) 23:2888–93. doi: 10.1016/j.jstrokecerebrovasdis.2014.07.018 25440367

[3] Palaniswami M Yan B. Mechanical thrombectomy is now the gold standard for acute ischemic stroke: implications for routine clinical practice. Interv Neurol. (2015) 4:18–29. doi: 10.1159/000438774, PMID: 26600793 PMC4640090

[4] Waqas M Rai AT Vakharia K Chin F Siddiqui AH. Effect of definition and methods on estimates of prevalence of large vessel occlusion in acute ischemic stroke: a systematic review and meta-analysis. J Neurointerv Surg. (2020) 12:260–5. doi: 10.1136/neurintsurg-2019-015172, PMID: 31444289

[5] Waqas M Mokin M Primiani CT Gong AD Rai HH Chin F. Large vessel occlusion in acute ischemic stroke patients: a dual-center estimate based on a broad definition of occlusion site. J Stroke Cerebrovasc Dis. (2020) 29:104504. doi: 10.1016/j.jstrokecerebrovasdis.2019.104504, PMID: 31761735

[6] Sjöstrand C Klironomos S. HISS–hemorrhagic and ischemic stroke study. Stockholm: (2019).

[7] Mazya MV Berglund A Ahmed N von Euler M Holmin S Laska AC. Implementation of a prehospital stroke triage system using symptom severity and teleconsultation in the Stockholm stroke triage study. JAMA Neurol. (2020) 77:691–9. doi: 10.1001/jamaneurol.2020.0319, PMID: 32250423 PMC7136864

[8] WHO. International Statistical Classification of Diseases and Related Health Problems. Tenth Revision (ICD-10). Available online at: http://www.who.int/classifications/icd/en/ (accessed September 19, 2022).

[9] Rai AT Seldon AE Boo S Link PS Domico JR Tarabishy AR. A population-based incidence of acute large vessel occlusions and thrombectomy eligible patients indicates significant potential for growth of endovascular stroke therapy in the USA. J NeuroInterv Surg. (2017) 9:722–6. doi: 10.1136/neurintsurg-2016-012515, PMID: 27422968 PMC5583675

[10] Saini V Guada L Yavagal DR. Global epidemiology of stroke and access to acute ischemic stroke interventions. Neurology. (2021) 97:S6–S16. doi: 10.1212/WNL.0000000000012781, PMID: 34785599

[11] Mokin M Pendurthi A Ljubimov V Burgin WS Siddiqui AH Levy EI. ASPECTS, large vessel occlusion, and time of symptom onset: estimation of eligibility for endovascular therapy. Neurosurgery. (2018) 83:122–7. doi: 10.1093/neuros/nyx352, PMID: 29106687

[12] Demeestere J Garcia-Esperon C Lin L Bivard A Ang T Smoll NR. Validation of the National Institutes of Health Stroke Scale-8 to detect large vessel occlusion in ischemic stroke. J Stroke Cerebrovasc Dis. (2017) 26:1419–26. doi: 10.1016/j.jstrokecerebrovasdis.2017.03.020, PMID: 28457621

[13] Beumer D Mulder MJHL Saiedie G Fonville S van Oostenbrugge RJ van Zwam WH. Occurrence of intracranial large vessel occlusion in consecutive, non-referred patients with acute ischemic stroke. Neurovasc Imaging. (2016) 2:11. doi: 10.1186/s40809-016-0022-5

[14] Hansen CK Christensen A Ovesen C Havsteen I Christensen H. Stroke severity and incidence of acute large vessel occlusions in patients with hyper-acute cerebral ischemia: results from a prospective cohort study based on CT-angiography (CTA). Int J Stroke. (2015) 10:336–42. doi: 10.1111/ijs.12383, PMID: 25319377

[15] Smith WS Lev MH English JD Camargo EC Chou M Johnston SC. Significance of large vessel intracranial occlusion causing acute ischemic stroke and TIA. Stroke. (2009) 40:3834–40. doi: 10.1161/STROKEAHA.109.561787, PMID: 19834014 PMC2796543

[16] Nicholls JK Ince J Minhas JS Chung EML. Emerging detection techniques for large vessel occlusion stroke: a scoping review. Front Neurol. (2021) 12:780324. doi: 10.3389/fneur.2021.780324, PMID: 35095726 PMC8796731

[17] Ding M Ebeling M Ziegler L Wennberg A Modig K. Time trends in atrial fibrillation-related stroke during 2001-2020 in Sweden: a nationwide, observational study. Lancet Reg Health Europe. (2023) 28:100596. doi: 10.1016/j.lanepe.2023.100596, PMID: 37180742 PMC10173271

[18] Duloquin G Béjot Y. Nationwide projections of ischemic stroke with large vessel occlusion of the anterior circulation by 2050: Dijon stroke registry. Front Public Health. (2023) 11:1142134. doi: 10.3389/fpubh.2023.1142134, PMID: 37304110 PMC10248396

[19] Rai AT Link PS Domico JR. Updated estimates of large and medium vessel strokes, mechanical thrombectomy trends, and future projections indicate a relative flattening of the growth curve but highlight opportunities for expanding endovascular stroke care. J Neurointerv Surg. (2023) 15:e349–55. doi: 10.1136/jnis-2022-019777, PMID: 36564202 PMC10803998

[20] Acciarresi M De Luca P Caso V Agnelli G D'Amore C Alberti A. Acute stroke symptoms: do differences exist between sexes? J Stroke Cerebrovasc Dis. (2014) 23:2928–33. doi: 10.1016/j.jstrokecerebrovasdis.2014.07.044, PMID: 25440370

[21] Owais SB Bulwa ZB Ammar FE. Differences in stroke clinical presentation among sexes. J Stroke Cerebrovasc Dis. (2024) 33:107807. doi: 10.1016/j.jstrokecerebrovasdis.2024.107807, PMID: 38851548

[22] Zürcher E Richoz B Faouzi M Michel P. Differences in ischemic anterior and posterior circulation strokes: a clinico-radiological and outcome analysis. J Stroke Cerebrovasc Dis. (2019) 28:710–8. doi: 10.1016/j.jstrokecerebrovasdis.2018.11.016, PMID: 30501979

[23] Frid P Drake M Giese AK Wasselius J Schirmer MD Donahue KL. Detailed phenotyping of posterior vs. anterior circulation ischemic stroke: a multi-center MRI study. J Neurol. (2020) 267:649–58. doi: 10.1007/s00415-019-09613-5, PMID: 31709475 PMC7035231

[24] Higgins HM Chen L Ravare BC Jeppson KA Bina HT Herson PS. Sex differences in acute ischemic stroke presentation are a matter of infarct location. Am J Emerg Med. (2023) 74:95–9. doi: 10.1016/j.ajem.2023.09.046, PMID: 37802001 PMC10843056

[25] Edzie EK Dzefi-Tettey K Gorleku P Amankwa AT Idun E Brakohiapa EK. Evaluation of the anatomical locations of stroke events from computed tomography scan examinations in a tertiary facility in Ghana. Cureus. (2021) 13:e14097. doi: 10.7759/cureus.14097, PMID: 33907641 PMC8065308

[26] Pirson F Boodt N Brouwer J Bruggeman AAE Hinsenveld WH Staals J. Etiology of large vessel occlusion posterior circulation stroke: results of the MR CLEAN registry. Stroke. (2022) 53:2468–77. doi: 10.1161/STROKEAHA.121.038054, PMID: 35543130

[27] Silva GS Lima FO Camargo EC Smith WS Lev MH Harris GJ. Gender differences in outcomes after ischemic stroke: role of ischemic lesion volume and intracranial large-artery occlusion. Cerebrovasc Dis. (2010) 30:470–5. doi: 10.1159/000317088, PMID: 20733301 PMC2992642

[28] van der Meij A Holswilder G Bernsen MLE van Os HJ Hofmeijer J Spaander FH. Sex differences in clot, vessel and tissue characteristics in patients with a large vessel occlusion treated with endovascular thrombectomy. Eur Stroke J. (2024) 9:600–12. doi: 10.1177/23969873241231125, PMID: 38420950 PMC11418468

[29] Béjot Y Daubail B Jacquin A Durier J Osseby GV Rouaud O. Trends in the incidence of ischaemic stroke in young adults between 1985 and 2011: the Dijon stroke registry. J Neurol Neurosurg Psychiatry. (2014) 85:509–13. doi: 10.1136/jnnp-2013-306203 24249786

[30] Bhayana K Handshoe JW Li Y Thompson NR Kharal M Saleem H. Effect of stroke etiology on treatment-related outcomes in young adults with large vessel occlusion: results from a retrospective cohort study. J Stroke Cerebrovasc Dis. (2024) 33:108027. doi: 10.1016/j.jstrokecerebrovasdis.2024.108027, PMID: 39307210

[31] Eriksson M Åsberg S Sunnerhagen KS von Euler M. Sex differences in stroke care and outcome 2005–2018: observations from the Swedish stroke register. Stroke. (2021) 52:3233–42. doi: 10.1161/STROKEAHA.120.033893, PMID: 34187179

[32] Riksstroke Årsrapport (2023) The Riksstroke annual report of 2023. Available online at: https://www.riksstroke.org (accessed December 20, 2024).

